# Protein S drives oral squamous cell carcinoma tumorigenicity through regulation of AXL

**DOI:** 10.18632/oncotarget.14753

**Published:** 2017-01-19

**Authors:** Ghada Abboud-Jarrous, Shivam Priya, Avi Maimon, Stuart Fischman, Mayan Cohen-Elisha, Rakefet Czerninski, Tal Burstyn-Cohen

**Affiliations:** ^1^ Institute for Dental Sciences, Faculty of Dental Medicine, The Hebrew University of Jerusalem, Israel; ^2^ Department of Oral Medicine, The Hebrew University - Hadassah School of Dental Medicine, Jerusalem, Israel

**Keywords:** protein S, PROS1, OSCC, AXL, squamous cell carcinoma

## Abstract

The TAM family of proto-oncogenic receptor protein tyrosine kinases, comprising of TYRO3, AXL, and MERTK, is implicated in many human cancers. Their activation leads to cancer cell proliferation, enhanced migration, invasion, and drug resistance; however how TAMs are activated in cancers is less understood. We previously showed that Protein S (PROS1) is a ligand of the TAM receptors. Here we identify PROS1 as a mediator of Oral Squamous Cell Carcinoma (OSCC) in proliferation, cell survival and migration. We demonstrate that excess PROS1 induces OSCC proliferation and migration. Conversely, blocking endogenous PROS1 expression using shRNA significantly inhibits cell proliferation and migration in culture. This inhibition was rescued by the addition of purified PROS1. Moreover, PROS1 knockdown reduced anchorage-independent growth *in-vitro*, reduced tumor xenograft growth in nude mice and altered their differentiation profile. Mechanistically, we identify the downregulation of *AXL* transcripts and protein following PROS1 knockdown. Re-introducing PROS1 rescues AXL expression both at the protein and transcriptional levels. The anti-proliferative effect of the AXL inhibitor R428 was significantly reduced following PROS1 inhibition, indicating the functional significance of PROS1-mediated regulation of AXL in OSCC. Taken together, we identify PROS1 as a driver of OSCC tumor growth and a modulator of AXL expression. Our results point to *PROS1* as a potential novel anti-cancer therapeutic target.

## INTRODUCTION

Head and neck cancer (HNSCC) is the sixth most common cancer worldwide; however the molecular mechanisms underlying HNSCC tumorigenesis are not well understood, impeding on its early detection and identification of candidate molecules for targeted therapy. The overexpression and hyperactivation of members of the TAM family of receptor tyrosine kinases – comprising TYRO3, AXL and MERTK is documented in many cancers [1]. Recently, the functional significance for overexpression of *AXL* and *MERTK* was reported for HNSCC, [2, 3]. More specifically, AXL was identified as a potential therapeutic target in oral squamous cell carcinoma (OSCC) [4], Head and Neck squamous cell carcinoma (HNSCC) [5] and esophageal cancer [6], with poor prognosis correlated to high *AXL* expression.

Signaling through AXL activates several intracellular pathways, leading to increased proliferation, enhanced migration, invasion and cell survival. Recent work identifies AXL overexpression to underlie the induction of alternative survival pathways leading to therapeutic resistance [3, 6–8]. The role of the TAM cognate ligands GAS6 and Protein S (PROS1) was demonstrated in homeostatic regulation of the immune, reproductive, vascular and nervous systems [9–16]. In cancer settings, the activation of TAM receptors by GAS6 was shown in several tumor models [17–21], however the role of PROS1 in oncogenic signaling and tumor biology has not been extensively investigated. We recently identified PROS1 as a TAM ligand in the mouse retina [11], which prompted us to investigate the role of PROS1 in TAM-mediated tumorigenesis.

Here, we show for the first time that PROS1 is highly expressed in OSCC cell lines SCC1 and SCC25, and provide evidence that PROS1 supports cancer cell proliferation and migration. Inhibition of PROS1 expression suppressed tumor cell proliferation, migration and anchorage-independent growth *in-vitro*. Reciprocally, cell proliferation was stimulated in the presence of PROS1. *In-vivo*, PROS1 inhibition decreased tumor growth and decreased tumor cell differentiation in a xenograft model. Finally, we identify PROS1 as a potent regulator of AXL expression, affecting AXL-mediated cell growth and migration. AXL transcripts and protein levels were down-regulated following PROS1 knockdown, and both were recovered upon incubation with PROS1. In keeping with PROS1-regulated AXL expression, sensitivity to the small molecule AXL inhibitor R428 was lost following PROS1 knockdown. Taken together, these results identify a novel role for PROS1 in OSCC, and advocate PROS1 as a potential therapeutic target in this disease.

## RESULTS

### PROS1 is highly expressed in oral squamous cell carcinoma cell lines

**Figure 1:**
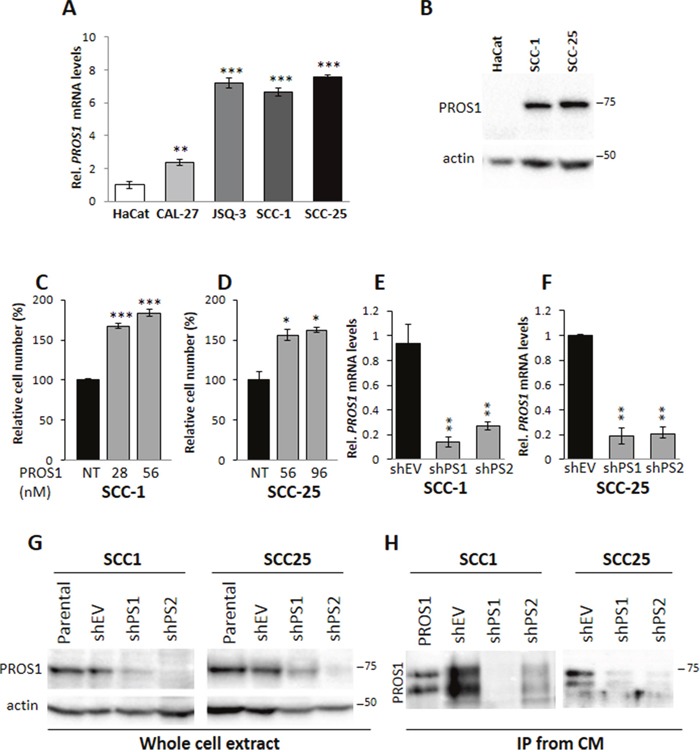
PROS1 is expressed in OSCC cells and stimulates cell proliferation **A**. Analysis of *PROS1* mRNA levels by realtime qPCR in different OSCC cell lines. Results presented are relative to *PROS1* mRNA levels in HaCat immortalized human keratinocytes. Graphs represent mean ± SEM from 3 independent experiments. ***P<0.001. **B**. Analysis of PROS1 protein levels in whole cell extracts from the indicated cell lines. High PROS1 levels are detected in SCC-1 and SCC-25 OSCC cell lines, but not in the immortalized human Keratinocyte cell line HaCaT. Actin serves a as loading control. One representative blot of three independent experiments is shown. **C, D**. Dose dependent effects of PROS1 on proliferation of SCC-1 (C) and SCC-25 (D) cells. hPROS1 was added to the cells at the indicated concentrations (nmol/L) 48 hours before performing proliferation assays. Proliferation is plotted as a percentage of growth relative to vehicle-treated cells. The means ± SEM of a representative experiment out of four are shown. *P<0.05, ***P<0.001. **E, F**. Effective knockdown of *PROS1* in SCC1 (E) and SCC-25 (F) OSCC lines by two different sh targeting sequences. RT-qPCR detection of *PROS1* mRNA in control (EV)-treated and PROS1-knockdown populations using two different PROS1- targeting sequences (shPS1, shPS2). qPCR data are normalized to β-actin. Graphs represent mean ± SEM from 3 experiments. **P<0.01. **G**. Analysis of PROS1 protein levels in whole cell extracts by western blot analysis in SCC-1 (left) and SCC-25 (right) parental, control-treated (shEV) and stable *PROS1-* knockdown cell lines (shPS1, shPS2). Actin serves as a loading control. Results are representative of five independent experiments. **H**. Analysis of secreted PROS1 levels from conditioned medium (CM) by immunoprecipitation (IP) followed by western blot with anti-PROS1 antibody from control and sh-PS stable cell lines. Purified PROS1 served as a positive control. Purified and secreted PROS1 appear as a doublet, following proteolytic cleavage by serum enzymes. Results are representative of five independent experiments.

To assess whether PROS1 may have a putative role in OSCC, we first evaluated *PROS1* expression by different OSCC cell lines. We found highest levels of PROS1 mRNA transcripts in SCC-1, SCC-25 and JSQ-3 cell lines, followed by CAL-27. PROS1 transcripts were barely detectable in HaCaT cells, an immortalized human Keratinocyte cell line (Figure 1A). SCC-1 and SCC-25 cells also expressed high PROS1 protein levels (Figure 1B). Moreover, analysis of the Oncomine public database (www.oncomine.org) revealed the O'Donnell Oral database [22], which showed significant overexpression of *PROS1* mRNA in cell lines from OSCC, especially from the tongue, sharing the same origin as SCC-1 and SCC-25 (Supplementary Figure 1). These results suggest that PROS1 may be a marker for OSCC and may play a role in the development of this cancer, particularly in the tongue. We therefore focused on SCC-1 and SCC-25 cell lines.

Since PROS1 was recently shown to function as a TAM agonist *in-vivo* [10, 11, 15] and *AXL* and *MERTK* hyperactivation to drive OSCC cell growth and migration [2, 4], we asked whether the overexpression of PROS1 in SCC-1 and SCC-25 may be functionally relevant in OSCC. To address this, we stimulated OSSC cells with exogenous PROS1, and measured cell growth. Culturing SCC-1 cells in the presence of 28 nmol/L PROS1 for 48 hours stimulated cell growth by 67% (P=0.0006) compared to non-stimulated cells (Figure 1C). Similar to SCC-1, addition of PROS1 (56 nmol/L) to SCC-25 cells stimulated their growth by 56% (P=0.02) (Figure 1D), indicating that PROS1 promotes proliferation of OSCC cells.

### *PROS1* inhibition in OSCC cell lines attenuates cell proliferation

To evaluate the functional significance of PROS1 expression in these OSCC cell lines we introduced stable shRNA constructs targeting *PROS1* mRNA into SCC-1 and SCC-25 cell lines following lentiviral infections. Five sh*PROS1* sequences were initially tested for their ability to inhibit *PROS1* expression. Four of these reduced *PROS1* transcript and protein levels with strongest inhibition by more than 85% as observed by RT-qPCR and western blot, respectively. Two stable puromycin-resistant knockdown lines shPS1 and shPS2, and two control lines using a non-relevant (sh*GFP*) or empty targeting vector (shEV) were generated for both parental SCC-1 and SCC-25 cell lines (Figure 1E–1F). Fresh knockdown infections were made periodically to avoid clonal effects. Compared to the parental and control-treated cells, PROS1 protein was significantly reduced in the cell lysates of knockdown lines (Figure 1G). Consistent with PROS1 being a secreted protein, we detected PROS1 by immunoprecipitation from the conditioned medium of control, but not of *PROS1*-targeted lines (Figure 1H).

**Figure 2:**
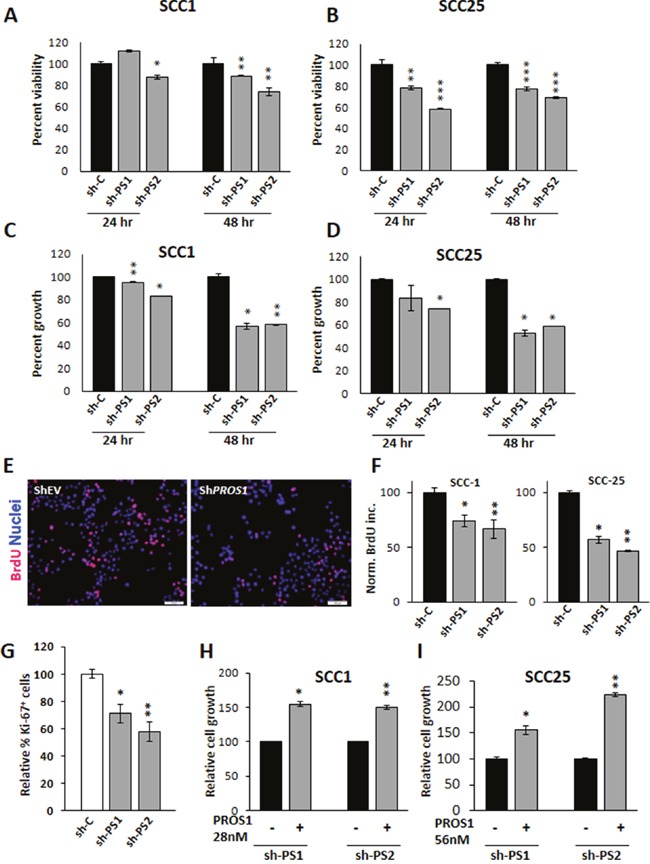
PROS1 kd affects cell viability and proliferation **A, B**. PROS1 knockdown affects cell viability. Analysis of cell viability following PROS1-kd by two different targeting sequences in SCC-1 (A) and SCC-25 (B) cells by the XTT assay. 3,000 cells were seeded in 96 wells, and assayed at the indicated times. Each time point represents the mean ±SEM of at least three replicates. Results are representative of five independent experiments. *P<0.05, **P<0.01; ***P< 0.001. **C, D**. PROS1 knockdown inhibits OSCC proliferation. Proliferation of control-treated and PROS1-kd SCC-1 (C) and SCC-25 (D) cells as measured at 24 and 48 hours post seeding. Proliferation was measured by crystal violet absorbance, and is presented as relative growth compared to growth of EV-treated cells. Each time point represents 6 replicates in three independent experiments. *P<0.05, **P<0.01. **E**. BrdU immunohistochemistry (red) indicating decreased BrdU incorporation in PROS1-kd SCC-1 cells compared to shEV. Nuclei are stained with Hoechst (blue). Results are representative of five independent experiments. **F**. Quantification of BrdU incorporation following PROS1 knockdown in SCC-1 (left) and SCC-25 (right) cell lines. Results are presented as % of BrdU+ cells. Each experiment was performed in N=4 replicates. Five different fields were documented and scored per each condition in every experiment. Results are representative of five independent experiments. *P<0.05, **P<0.01. **G**. Quantification of Ki-67+ cells. Percent Ki-67+ cells in SCC-25 cells (# Ki-67^+^/ # Hoechst^+^ nuclei), normalized to control-treated cells. Each experiment was performed in triplicate, and at least five different fields were scored per condition. Mean values ± SEM are shown from at least three different experiments. *P=0.018; **P= 0.0059. **H, I**. Exogenous PROS1 rescues the growth rates of PROS1-kd SCC-25 and PROS1-kd SCC-1 cells. PROS1 (28 nmol/L for SCC-1 and 56 nmol/L for SCC-25) was added to the growth medium. Proliferation was measured 48 hours later by crystal violet absorbance, and is presented as relative growth compared to PROS1-kd cells without PROS1. Every experiment was performed in triplicates or sextuplicates. Results are representative of four independent experiments. *P<0.05, **P<0.01.

To determine whether PROS1 contributes to OSCC oncogenic phenotypes, we first studied the effects of *PROS1* inhibition on cell growth and proliferation. Cell proliferation was evaluated using the XTT and crystal violet methods. Knockdown of *PROS1* in both OSCC cell lines decreased their proliferation rate in culture, compared with control-treated cells. Decreased cell viability was observed for both sh1 and sh2 knockdown constructs in SCC-1 and SCC-25 cells (Figure 2A, 2B). Similarly, measuring cell growth by crystal violet also revealed reduced cell numbers 24 and 48 hours after plating in sh1 and sh2 PROS1-kd lines, compared to control-treated cells (Figure 2C, 2D). To directly assess whether proliferation rates are affected following PROS1 ablation we monitored and compared the percent cells that have incorporated BrdU (mitotic index) as a function of *PROS1* expression. PROS1 inhibition using both sh1 and sh2 constructs reduced the mitotic index of SCC-1 cells by 26% (P = 0.04) and by 33% (P = 0.01), respectively. Similarly, PROS1 knockdown in SCC-25 cells reduced the proliferative index by 43% (P = 0.003) and by 53% (P < 0.0001), indicating an inhibitory effect on the proliferation (Figure 2E, 2F). Similar results were obtained following transient *PROS1* inhibition using the siRNA method. SCC-25 cells were transfected with a pool of four OnTarget siRNAs, resulting in 83% inhibition of *PROS1* transcripts (P<0.0001). PROS1 protein levels were also inhibited (Supplementary Figure 2A, 2B). As for sh-treated cell lines, we found that si-mediated targeting of PROS1 reduced cell growth by 31% (P=0.007) and inhibited BrdU incorporation rates by 42% (P=0.01) (Supplementary Figure 2C, 2D). Fewer cycling cells were also measured by Ki-67 immunoreactivity in cultured SCC-25 shPS1 and shPS2 cells, compared to control-treated cells (30%; P=0.018 and 45% inhibition; P=0.0059, respectively) presented in Figure 2G.

To test the direct dependence of SCC-1 and SCC-25 cell proliferation on PROS1, we hypothesized that addition of PROS1 to the growth medium should rescue the inhibited cell growth observed in sh-treated lines. Indeed, addition of PROS1 to the growth medium reversed the growth-arrest observed in both SCC-1 and SCC-25 PROS1-kd lines. Supplementation of PROS1 increased proliferation rates of SCC-1-shPS1 and shPS2 lines by 54% (P = 0.004) and 50% (P = 0.001), respectively (Figure 2H). Similarly, proliferation of SCC-25 sh1 and sh2 lines had increased by 55% (P = 0.02) and by over 100% (P = 0.001) following addition of PROS1 to the growth medium (Figure 2I). Taken together, our data show that PROS1 is highly expressed in OSCC cells and that the KD of PROS1 reduces cell proliferation, a phenotype that can be rescued by the addition of exogenous PROS1.

### *PROS1* knockdown leads to decreased OSCC cell migration

**Figure 3:**
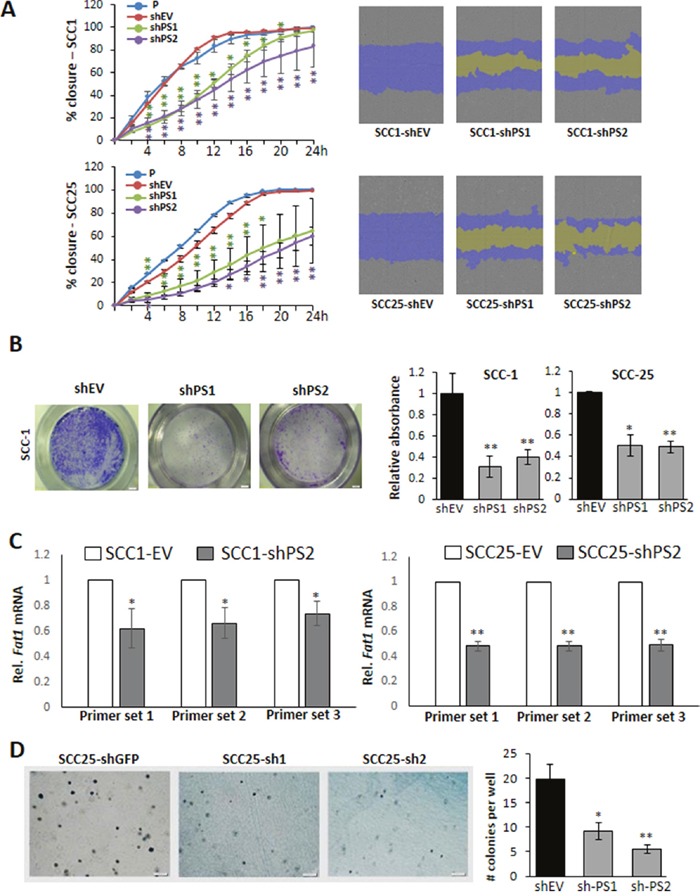
PROS1 inhibition attenuates migration and growth in soft agar **A**. Quantification of the scratch closure area as percent of the original scratch area is plotted against time (left) and the actual images of the scratch area (right) as automatically performed by the IncuCyte cell ZOOM live cell analysis system. Representative scratches are shown on the right: covered wound areas are depicted in purple, uncovered areas in yellow. Inhibition of PROS1 attenuated migration in both SCC-1 (top) and SCC-25 (bottom) cells. Results are representative of five experiments, each in triplicate or sextuplicates using different batches of cells. P values are indicated by asterisks for each time point on the graphs. * P<0.05; **P<0.01. **B**. *PROS1* knockdown inhibits cell migration in a transwell migration assay. Cells were allowed to migrate for 4 hours, stained and documented. Representative transwells are shown for SCC-1 lines after staining the cells that have migrated with crystal violet (left). Quantification of migratory cells was performed using crystal violet (right). Data represent means ± SEM from at least three independent experiments using different batches of cells (N≥15 in 4 independent experiments). *P<0.05, **P<0.05. **C**. *FAT1* expression is reduced in OSCC lines following *PROS1*-knockdown. Relative *FAT1* mRNA levels were normalized to *GAPDH* and evaluated using primers targeting three different locations spanning the large transcript to assess expression of the full transcript [23]. Representative data from one of three independent experiments are shown for each cell line. *P<0.05; **P<0.01. **D**. Anchorage-independent growth is attenuated following PROS1-knockdown. Soft agar growth assay showing colony formation 14 days after seeding. Representative colony growth is shown. Quantification of anchorage-independent colony formation in control (shGFP) and following PROS1 knockdown using two different PROS1 targeting vectors is shown. Data represent means ± SEM from at least three independent experiments using different batches of cells (N=3 in 3 independent experiments). *P<0.05, **P<0.05.

To assess the effect of PROS1 knockdown on OSCC cell migration we first employed the wound healing/scratch assay. Automated scratches and live imaging of scratch closure was performed using the Incucyte Zoom system up to 24h. Control (shEV) cells closed the scratch at 14 and 18 hrs for SCC-1 and SCC-25, respectively. By contrast, SCC-1 cells stably transfected with two different shPROS1 vectors had only covered 63% (P = 0.0167) and 50% (P = 0.000196) of the scratch area, respectively, and SCC25-shPS1 and SCC25-shPS2 had 50% (P = 0.029) and 40% (P = 0.0087) of the scratch area closed (Figure 3A). We also measured cell migration employing the transwell system. The migration of sh*PROS1* SCC-1 cells was inhibited by 40% ± 2.3% compared to control shEV cells (P = 0.4). Similarly, the migration of sh*PROS1* SCC-25 cells was 62% ± 2.6% that of control cells (P=0.011), as measured by crystal violet staining of cells that have migrated to the bottom side of the insert (Figure 3B).

To gain a deeper mechanistic understanding on the effects of PROS1 on migration, we assessed the levels of the cadherin superfamily protein FAT1, which was shown to be important for cell mobility and invasion particularly in OSCC, also shown to be expressed by SCC-25 cells [23]. To cover different regions of the extremely large *hFAT1* transcript (14,773 bp) we used three sets of primers spanning different transcript regions to assess *FAT1* expression following PROS1-KD. All primer pairs revealed inhibition of *FAT1* mRNA in SCC-25 and SCC-1 PROS1-KD cells (Figure 3C). Together, our results indicate that inhibition of PROS1 decreases the migratory capacity of OSCC cells.

### PROS1 knockdown mitigates OSCC anchorage-independent growth

We next evaluated the effect of PROS1 knockdown on anchorage-independent growth in SCC-25 cell lines using the soft agar assay. Compared to shGFP, shPS1 and shPS2 cells developed significantly fewer colonies in soft agar after 14 days in culture. Colony numbers generated by shPS1 and shPS2 decreased by 54% (P = 0.019) and 72% (P = 0.005), respectively (Figure 3D).

### Reduction of tumor growth *in-vivo* following PROS1 knockdown

**Figure 4:**
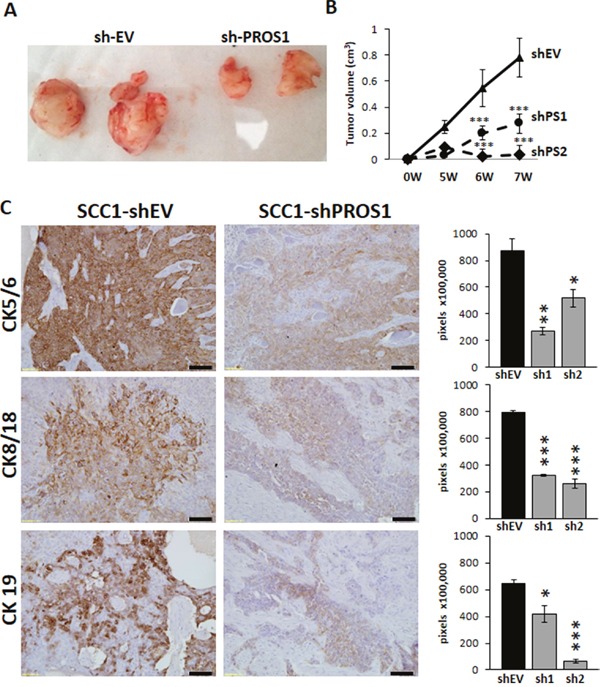
Inhibition of PROS1 in OSCC inhibits tumor growth in a xenograft model **A**. Representative tumors of SCC-25 treated with shEV or sh-PROS1 seven weeks after subcutaneous injection. **B**. Dynamics of tumor growth. Tumor volume was measured weekly and is plotted in cm^3^ against time (weeks). Inhibition of PROS1 resulted in significant tumor growth retardation. ***P<0.001. Eight tumors were induced per SCC1-EV, shPS1 and shPS2, injected bilaterally into four mice in the flank region. **C**. The tumor markers Cytokeratins 5/6, 8/18, 19 are downregulated in PROS1-KD xenografts. Representative images are shown, following immune-detection. Quantification of immunostaining indicates significant downregulation of the cytokeratins CK5/6, CK8/18 and CK19 - all markers of tumor aggressiveness to correlate with inhibition of PROS1. Mean values ± SEM are shown for at least three different tumors. Images were quantified using ImagePro software. *P<0.05, **P<0.01; ***P< 0.001.

To determine whether PROS1 inhibition could also constrain OSCC tumorigenic potential *in-vivo*, SCC1 cells were injected subcutaneously into the flanks of immune-deficient nude mice. At 7 weeks post implantation PROS1 knockdown tumors had significantly smaller tumor volumes with 66% smaller tumors for shPS1 cells (27 mm^3^ mean tumor volume, P < 0.03), and 96.2% smaller tumors for shPS2 (3 mm^3^ mean tumor volume, P < 0.001), compared to shEV (78 mm^3^ mean tumor volume) (Figure 4A, 4B). Two injection foci of shPS2 did not develop tumors by the end of the experiment.

Sections of shEV, shPS1 and shPS2 were next subjected to pathological evaluation for the degree of differentiation in the various tumors. Differentiation was scored by a modification of the conventional grading system described by Anneroth, et al. [24]. While 100% (6/6) of shEV tumors scored as poorly differentiated, as did 100% (8/8) tumors of the parental SCC1 cell line, only 5 of 8 (62.5%) shPS1 and 1/6 (16.6%) tumors of shPS2 received this score. Among shPS1 tumors, 66% (4/6) were graded as moderate-to-well differentiated and 16.6% (1/6) was graded either as poorly or poorly-to-moderately differentiated. SCC1-shPS2 tumors also exhibited a higher degree of differentiation, with 2/8 (25%) tumors moderately differentiated, 1/8 (12.5%) poorly-to-moderately differentiated.

To allow further molecular identification and quantification of the tumor cells as a function of PROS1 expression, we assessed the expression levels of cytokeratins (CK) 5/6, 8/18 and 19 by immunohistochemical analysis. Elevated levels of CK 5/6, CK 8/18 and CK 19 in OSCC patients are associated with increased dysplasia, are correlated with acquisition of invasive growth properties, and are considered markers for poor clinical outcome [25–29]. Remarkably, compared to shEV, both shPS1 and shPS2 tumors exhibited a significant reduction in expression levels of these cytokeratins. SCC1-shPS1 and shPS2 tumors had 69% (P=0.003) and 40% (P=0.029) lower expression of CK5/6. CK 8/18 levels were 56% (P=0.0005) and 66% (P=0.0001) lower than shEV tumors, and CK19 levels were 35% (P=0.0001) and 89% (P=0.027) lower, respectively (Figure 4C).

We verified PROS1 expression in shEV tumors, as well as conservation of PROS1 knockdown in shPS1 and shPS2 tumors during *in-vivo* growth by immunohistochemistry of tumor sections at the endpoint. PROS1 immunoreactivity was observed in shEV in the basal and intermediate cell layers, with a strong signal in the basal cells surrounding the tumor nest structures characteristic of OSCC. Only faint PROS1 signal was obtained in shPS tumors (Supplementary Figure 3A-3D). The marked reduction in cytokeratin levels following PROS1 knockdown supports the pathological assessment, and together with reduced tumor growth *in-vivo* indicate decreased malignancy of OSCC tumors following PROS1 knockdown.

We next assessed cell proliferation in the xenograft tumors by Ki-67 immunoreactivity. Ki-67 expression is correlated with cell proliferation and is a prognostic marker for various cancers. Compared to control-treated xenografts, shPS1 tumors showed lower proliferative activity (P=0.042; Supplementary Figure 4A), in agreement with lower BrdU and Ki-67 levels *in vitro* (Figure 2E–2G). The lower proliferation rates in tumors inhibited for PROS1 expression also showed more numerous apoptotic foci, and contained more apoptotic cells positive for DNA fragmentation, as measured by the TUNEL (Terminal deoxynucleotidyltransferase –mediated dUTP Nick End Labeling) reaction (P=0.024 for shPS1; P=0.001 for shPS2; Supplementary Figure 4B, 4C). Apoptotic foci are also evident by the numerous pyknotic nuclei, with condensed heterochromatin (Supplementary Figure 4B, 4C). Taken together, we conclude that PROS1 knockdown increases apoptotic cell death in tumors, which is also reflected by fewer cycling Ki-67-positive cells.

### Regulation of AXL expression by PROS1

**Figure 5:**
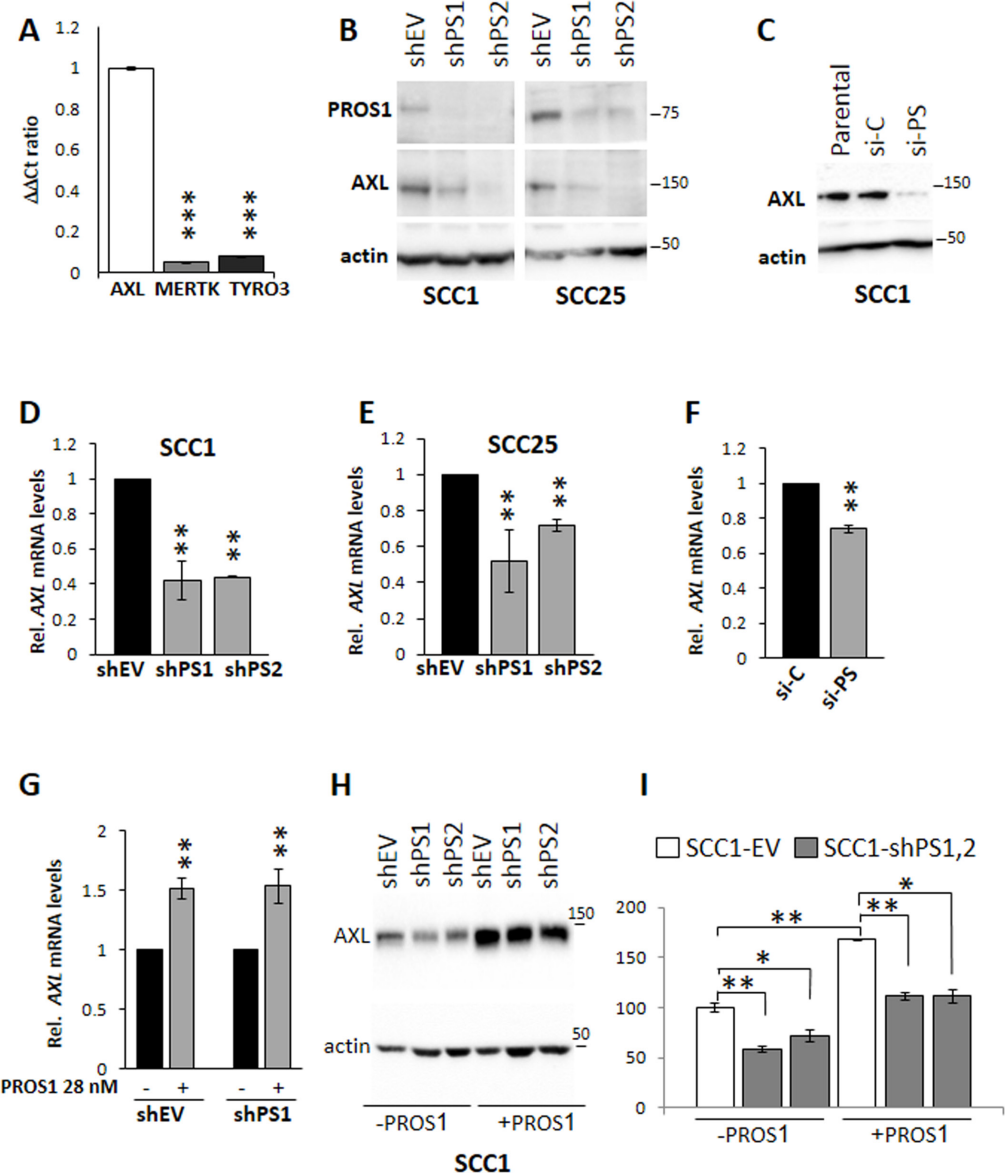
PROS1 regulates AXL expression in OSCC **A**. AXL is the dominant TAM receptor expressed in SCC-1 cells. Expression of TYRO3, AXL and MERTK are shown relative to actin. Realtime qPCR results are presented as ΔΔCt ratio normalized to AXL, and are representative of four independent experiments. ***P<0.0001. **B**. Reduced AXL protein levels following sh-mediated knockdown of *Pros1* in SCC-1 (left) and SCC-25 (right) cells. PROS1 and AXL protein levels are shown as detected by western blot in the different sh-treated cell lines. Actin serves as a loading control. Results are representative of three independent experiments. **C**. AXL protein levels are significantly reduced after si-PROS1 interference (si-PS) in SCC1 cells, but not following control si treatment (si-C), as shown by western blot. Actin is a loading control. Results are representative of two independent experiments. **D, E**. *PROS1* knockdown affects AXL transcription. RT-qPCR detection of *AXL* mRNA in SCC-1 (D) and in SCC-25 (E) control (EV)-treated and PROS1-knockdown populations using two different PROS1- targeting sequences (shPS1, shPS2). qPCR data are normalized to β-actin. Graphs represent mean ± SEM from 4 experiments. **P<0.01. **F**. Reduced *AXL* transcription following si-mediated inhibition of PROS1. Graphs represent mean ± SEM from 2 experiments. **P<0.01. **G**. PROS1 stimulates AXL transcription. SCC-1 shEV and shPS1 cells were grown either without or with supplementation of PROS1 (28 nmol/L) for 48 hours before RNA was extracted and assessed for AXL expression. Graphs represent mean ± SEM from 3 experiments. **P<0.01. **H**. AXL protein levels as seen by western blot of whole cell extracts are restored following exposure to exogenous PROS1. PROS1 (28 nmol/L) was added to the growth medium of treated cells (+PROS1) for 72 hours. Control cells were not supplemented with PROS1. Actin was used as a loading control. **I**. Quantification of AXL protein following PROS1 knockdown and rescue. Baseline levels of AXL protein band intensity in SCC1-EV cells were set as 100%. AXL protein levels decreased following knockdown in shPS1 (P=0.006) and shPS2 (P=0.026) lines. Culturing the cells in the presence of exogenous PROS1 (28 nmol/L) for 48 hours increased AXL levels in EV controls (P=0.001), and in shPS1 and shPS2, bringing them to levels of control cells without PROS1 supplementation (P= non-significant). AXL levels in the presence of PROS1 remained low in shPS1 (P=0.004) and shPS2 (P=0.019) compared to PROS1-treated EV controls. Band intensity was measured using the Bio-Rad ChemiDoc MP imaging system. Results were normalized to actin and the relative mean values ± SEM from two independent experiments are presented.

To reveal the molecular mechanism by which PROS1 contributes to tumorigenesis in OSCC, we hypothesized that PROS1 may function through the proto-oncogenic TYRO3, AXL and MERTK (TAM) family of receptor tyrosine kinases, known to bind PROS1 [9, 10]. We therefore examined the status of TAM receptor expression in our cells. In keeping with previous studies which identified high AXL expression and AXL-mediated oncogenic properties in OSCC and head and neck cancers [3, 4, 6], we found AXL to be highly expressed in our cell lines, whereas TYRO3 and MERTK levels were barely detectable (Figure 5A). Surprisingly, the downregulation of PROS1 by shRNA was associated with suppression of AXL protein levels as determined by western blot in both SCC-1 and SCC-25 cells (Figure 5B) and immunocytochemistry (Supplementary Figure 5D). Similar results were obtained by silencing PROS1 using siRNA (Figure 5C). To determine whether *PROS1* silencing affected AXL transcription, *AXL* mRNA levels were assessed following *PROS1* silencing using either shRNA or siRNA. Both approaches indicated that downregulation of PROS1 resulted in inhibition of *AXL* transcription (Figure 5D–5F). To confirm responsiveness of AXL expression to PROS1, we incubated shPS1 and shPS2 cells that have been silenced for *PROS1* expression with exogenous PROS1 protein, attempting to rescue *AXL* expression. Reintroducing PROS1 into the culture resulted in re-induction of *AXL* mRNA and protein levels, as assessed by RT-qPCR (P=0.001 for sh-EV; P=0.01 for shPS1) and western blot, respectively (Figure 5G–5I). PROS1 knockdown did not affect the expression of Tyro3, Mertk or Gas6 (Supplementary Figure 5A-5C) indicating AXL as the only TAM signaling component affected by PROS1-kd. Taken together, both silencing and rescue approaches identify AXL expression levels are regulated by PROS1.

**Figure 6:**
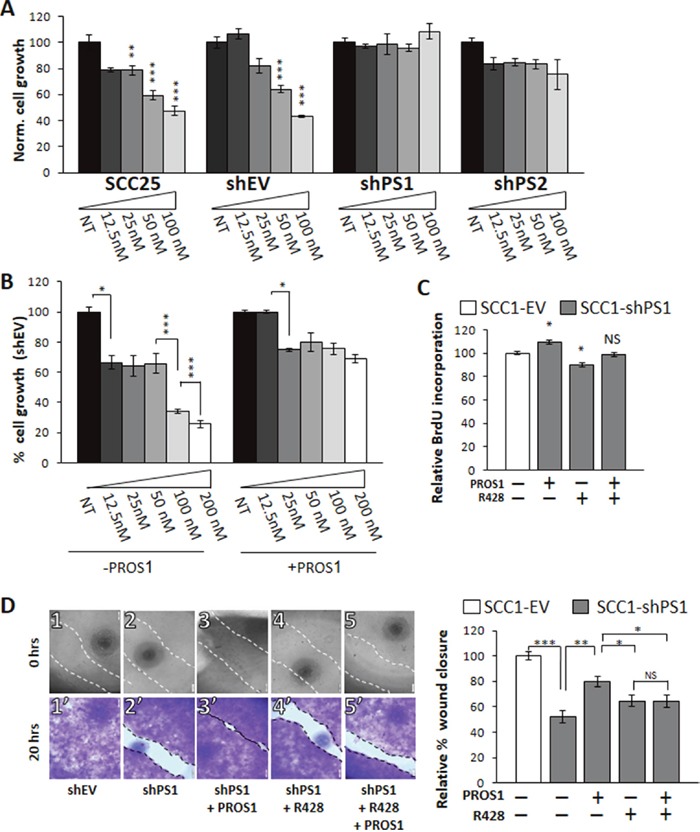
PROS1 modulates the sensitivity of OSCC to the AXL inhibitor R428 **A**. PROS1-KD cells are less sensitive to R428. SCC-25 parental, control (shEV), shPS1 and shPS2 cells were subjected to increasing doses of the AXL TKI R248 (12.5-100 nmol/L as indicated) for 48 hours before performing proliferation assays. Proliferation is plotted as a percentage of growth normalized to the number of cells seeded and relative to vehicle-treated cells using the crystal violet method. Parental and control-treated SCC25 cells showed a dose-dependent sensitivity to R428, whereas both sh-PROS1-treated lines lost AXL-mediated R428 sensitivity. The means ± SEM of a representative experiment out of three are shown. *P<0.05, **P<0.01, ***P<0.001. **B**. Exogenous PROS1 attenuates sensitivity to R428. SCC25-shEV cells were seeded as above, and grown in the presence of R428 dilutions either without or with 28 nmol/L PROS1 for 48 hours. Cell number is plotted as % growth normalized to the number of seeded cells and was detected using the crystal violet method. The means ± SEM of a representative experiment out of three are shown. *P<0.05. **C**. BrdU incorporation is affected by PROS1 levels. SCC1-shEV and shPS1cells were seeded as described above, and cultured in the presence or not of 28 nmol/L PROS1 and/or R428 (50 nmol/L) for 48 hours. The percent of BrdU+ cells (number of Brdu+ cells/number of nuclei) was calculated. Bars represent the relative mean ± SEM values of one experiment, representative of three independent experiments. *P<0.05, NS = not significant. **D**. Endogenously expressed PROS1 mediates migration of OSCC through AXL. The % wound area closed 20 hrs after generating the scratch is presented. Inhibition of endogenous PROS1 attenuates wound closure, which is rescued following incubation with PROS1 (28 nmol/L). Inhibition rates obtained for shPS cells that were treated with R428 (25 nmol/L) were similar to those following PROS1 knockdown alone. Scratch images are shown immediately after scratch generation (top panels), and after fixation and staining with crystal violet 20 hours later (lower panels) for each treatment, as indicated. Control-treated cells had closed 100% of the scratch area (1, 1′). By contrast, only 52% of the scratch area was closed by PROS1-sh1 cells (2, 2′). Addition of PROS1 (28 nmol/L) to the growth medium rescued scratch closure (3, 3′). The presence of R428 (25 nmol/L) inhibited scratch closure (4, 4′) and abolished the rescue potential of PROS1 (5′ 5′). Scratch closure measurements are presented as % wound closure. Bars depict the means ± SEM of three independent experiments, with N = 6 replicates in each experiment. ***P< 0.001; **P<0.01; *P<0.05, NS = no statistical significance.

To investigate the functional significance of PROS1-driven AXL expression, we treated parental and PROS1-knockdown cells with R428, a high affinity AXL-specific inhibitor [30]. R428 was previously shown to effectively block AXL phosphorylation and AXL-mediated cellular events, including cell migration, proliferation and angiogenesis in a variety of cancer cells, including Head and Neck Squamous Cell Carcinoma (HNSCC) [3, 31]. The proliferation of SCC-1 and SCC-25 parental, control (shEV) and shPS knockdown cells was measured following dose dependent R428 administration using the crystal violet method. R428 treatment (50 nmol/L) inhibited growth of the parental SCC-25 and control-treated SCC25-shEV cells by 40% (P=0.0008) and 36% (P=0.023), respectively (Figure 6A). By contrast, the growth rate of SCC25-shPS1 and SCC25-shPS2 in which AXL expression is downregulated was not affected by R428 treatment, even at a higher dose (100 nmol/L) (Figure 6A). These results indicate that OSCC cells have lost the AXL-mediated cell growth following PROS1 knockdown, and that this knockdown has functional significance.

We next aimed to directly evaluate whether the induced cell proliferation following stimulation by PROS1 described above (Figure 1C, 1D and Figure 2H, 2I) is mediated through AXL. For this, we cultured SCC25-shEV cells with or without exogenous PROS1, and treated the cells with increasing concentrations of R428. We hypothesized that if PROS1 induction of cell growth is AXL-dependent and if PROS1 positively upregulates AXL expression (Figure 5G–5I) then PROS1 addition may attenuate the toxic effect of R428. At the concentrations tested, SCC25-shEV control cells that were supplemented with PROS1 showed a decreased sensitivity to R428, compared with that of SCC25-shEV cells not supplemented with PROS1 (Figure 6B). Our results show that in the absence of exogenous PROS1, R428 toxicity and inhibition of cell growth is dose-dependent for R428 concentrations ranging from 25 nmol/L to 200 nmol/L. This effect, however, was lost after the cells were supplemented with PROS1 (Figure 6B).

Akin to cell growth and survival (Figure 6B), addition of exogenous PROS1 increased the BrdU incorporation rates in SCC1-shPS1 cells by 10% (P = 0.03), whereas R428 inhibited BrdU incorporation of SCC1-shPS1 by 11% (P = 0.022, Figure 6C). To understand whether PROS1 functions through AXL in stimulating cell proliferation, we attempted rescue with PROS1 in the presence of the AXL inhibitor R428. Inhibiting AXL abolished the growth stimulatory effect of PROS1 (P = not significant, Figure 6C), suggesting that PROS1 action is chiefly mediated by AXL.

We also tested whether the expression levels of cytokeratin 18 are responsive to PROS1 stimulation and AXL inhibition *in vitro*. Interestingly, the downregulation of cytokeratin protein observed in tumor sections (Figure 4C) was not mirrored *in vitro*: immunohistochemistry on cultured shPS lines detected comparable levels of CK14, CK18 and CK5 protein (Supplementary Figure 6A). This is attributed to the role of the cytokeratins as intermediate filaments which play major functional roles in epithelial tissues via cell-cell contact through desmosomes and hemidesmosomes as in stratified epithelium. This stratified feature is somewhat recapitulated in the xenograft tumors (e.g. the nested structures, Supplementary Figure 3), but lost in culture. Indeed, dissociation of the stratified epithelium and seeding was shown to induce changes in cytokeratin expression [32], indicating the regulation of keratin expression is associated to tissue cytoarchitecture [33]. The KRT18 mRNA levels of cultured SCC1-shPS cells were nevertheless responsive to PROS1 expression, and was downregulated in PROS1-kd cells (P=0.0002). Expression of KRT18 mRNA was rescued following addition of PROS1 (P=0.0096), but this rescue was attenuated in the presence of R428 (Supplementary Figure 6B). These results indicate the regulation of CK18 – a marker of OSCC tumor aggressiveness [26, 28] by PROS1 and AXL, and highlight the vital contribution of stratified tissue architecture for CK18 protein expression.

Finally, to assess whether the decreased migratory capacity following PROS1 inhibition (Figure 3) is also mediated through AXL, we assessed wound healing closure of SCC1-shPS1 cells in the presence of PROS1 or R428. In keeping with our previous results, inhibition of PROS1 attenuated closure of the scratch area (P<0.0001) (Figure 6D 1–2’). Moreover, addition of PROS1 to these PROS1-kd cells stimulated wound closure (P=0.001), rescuing the wound healing phenotype (Figure 6D 3-3’). Wound closure was similar between shPS1 cells and shPS1 cells that were treated with R428 (P = 0.137), suggesting PROS1 is an effective regulator of AXL-mediated migration. This is further stressed by the fact that addition of R428 eliminated the rescue effect of PROS1 (P = 0.157, Figure 6D 3-5’). Taken together, these results indicate that targeting PROS1 has a major impact on AXL expression and AXL-related cellular proliferation and migration.

## DISCUSSION

In this report, we reveal a fundamental and previously unknown role for PROS1 in OSCC malignancy. We identify high expression of PROS1, a ligand for the TAM receptors and a potent blood anticoagulant in OSCC cell lines, and show that endogenously expressed PROS1 is associated with multiple tumorigenic phenotypes. PROS1 stimulates proliferation and migration of OSCC cell lines and inhibition of PROS1 expression decreased proliferation, migration and anchorage-independent growth. Furthermore, knockdown of PROS1 reduced tumor growth in a xenograft transplantation model. Tumors inhibited for PROS1 expression appeared histologically more differentiated, and had downregulated expression of cytokeratins associated with increased tumor cell aggressiveness. We further reveal that PROS1 regulates the expression of AXL kinase, and demonstrate that PROS1-mediated cell growth and migration is AXL-dependent. Tumors inhibited for PROS1 expression were characterized by attenuated proliferation coupled with increased apoptotic foci, in agreement with AXL being a mediator of tumor cell survival [34]. High PROS1 levels were found to be correlated with high AXL levels in tongue SCC cell lines derived from patients (Supplementary Figure 1) [22]. Finally, the downregulation of AXL expression following PROS1 knockdown suggests therapeutic relevance to PROS1 inhibition.

There is considerable evidence linking the overexpression of TAM receptors in tumor cells, leading to increased proliferation, migration, survival and drug resistance in a multitude of cancers [1]. Among TAM family members, AXL overexpression is most prominent in various tumor models [34–37] and its inhibition was shown to reduce tumorigenesis and metastasis in several experimental models both in culture and in mice, including breast and lung [30, 31, 35, 38] Acute Myeloid Leukemia [39], head and neck and oral squamous cell carcinomas [3–6, 31, 35]. Thus, understanding the molecular regulation of AXL expression in tumor cells is of great importance. Consequently, AXL has become an attractive therapeutic target [36, 37, 40], leading to the development of AXL inhibitors, with several molecules in preclinical phases and R428 (licensed as BGB324) being the first small molecule AXL inhibitor tested in clinical trials [41].

We previously identified PROS1 as an *in-vivo* ligand for TAM receptors in the mouse eye, where it is important for the circadian uptake of photoreceptor outer segments by retinal pigment epithelium [11], mediated through MERTK [10]. PROS1 was further demonstrated to activate TAM signaling in the regulation of inflammation and immune response [15]. MERTK was recently found to be overexpressed in head and neck squamous cell carcinoma tumors (HNSCC), where its inhibition reduced the migratory potential of HNSSC cells [2]. Although we cannot rule out the involvement of TYRO3 and MERTK in mediating OSCC tumorigenicity, we believe this is less likely as their basal expression levels was barely detectable by real-time qPCR (Figure 5A), and expression of TYRO3, MERTK and GAS6 remained unchanged following PROS1-KD (Supplementary Figure 5). By contrast, we found AXL to be the predominantly expressed TAM receptor in our SCC-1 and SCC-25 cells (Figure 5A), as was also reported for additional OSCC cell lines [4], HNSCC cell lines and tumors [5].

The tumor-promoting properties of AXL can be stimulated by the TAM ligand GAS6, which binds to AXL with highest affinity [42] if co-expressed within tumor cells [18, 43–45]. Alternatively, GAS6 expressed by the tumor microenvironment was also shown to activate AXL in a paracrine manner [19, 46]. Indeed, activation of AXL by GAS6 was demonstrated in various cancer models [4, 17, 19, 20]. However, a ligand-independent mechanism was also demonstrated for AXL activation either by reactive oxygen species [47, 48], by homodimerization upon AXL overexpression [49, 50], or by heterodimerization with EGFR (epidermal growth factor receptor) as was shown in a breast cancer model [8]. Thus, in keeping with the cloning of AXL as a transforming gene overexpressed in myeloid leukemia [51] high expression levels of AXL may facilitate its ligand-independent activation. Although we cannot exclude the possibility that PROS1 directly stimulates AXL in the OSCC cell lines tested, this possibility seems less likely, as the binding of PROS1 to AXL in other cell types tested did not stimulate AXL phosphorylation and downstream signaling [10, 52]. It is tempting to speculate that PROS1-driven AXL expression may lead to functional receptor heterodimerization with EGFR, as was shown for HNSCC and esophageal squamous cell carcinoma [6]. Our results show that knockdown of PROS1 reduced AXL protein levels in the OSCC lines tested (Figure 5B, 5C), and that stimulation of these cells with PROS1 rescued AXL expression (Figure 5G–5I). Axl expression is also negatively regulated by the microRNAs (miR) miR-34 and miR-199a/b, with an inverse correlation between AXL protein levels and miR-34a expression found in a panel of cancer cell lines [53]. Interestingly, a meta-analysis study corroborated previous findings that decreased expression of miR-34a in HNSCC is associated with poor prognosis [54]. Akin to AXL, PROS1 expression was recently found to be inversely correlated to miR-34a in HepG2 hepatoma cells [55]. This report, together with our findings presented herein, raise the speculation whereby miR-34a and PROS1 possibly act synergistically to increase AXL expression: initially, low miR-34a expression leads to upregulated PROS1 [55] and AXL levels [53]. Subsequently, a parallel pathway is engaged in which the high levels of PROS1 further upregulate AXL transcription and protein (this paper). Together, the cumulative impact results in a feed-forward mechanism to increase AXL expression in tumor cells. However, further studies are needed to reveal the exact mechanisms by which PROS1 regulates AXL expression.

Our histologic analysis of *in-vivo* xenograft tumors reveals the expression of PROS1 by circumferential cells delineating the keratin nest-like structures (Supplementary Figure 3A, 3C), equivalent to the proliferative basal cell layer of the oral mucosal epithelium in human OSCC tumors, and consistent with high PROS1 expression shown to be confined to the epidermal basal layer in human skin [56]. Taken together, the normal expression of PROS1 in the epidermal basal layer, its high expression in the basal cell layer of OSCC xenograft tumors and its proliferative effect on OSCC cells (Figure 1C, 1D and Figure 2C–2I) suggest a role for PROS1 in the regulation of epithelial cell proliferation, and that dysregulated PROS1 expression may contribute to OSCC etiology. Accordingly, compared to control-treated cells, PROS1 knockdown cells appeared histologically more differentiated (Supplementary Figure 3A, 3B), and correlated with a reduction in cytokeratin markers CK5/6, CK8/18 and CK19 (Figure 4) associated with increased dysplasia and tumor aggressiveness.

In conclusion, our data indicate PROS1 is a potential therapeutic target in OSCC, preventing tumor cell proliferation and migration. PROS1 inhibition can be used to downregulate AXL levels. Combined inhibition of PROS1 and AXL may possibly allow lower dose treatment or provide additive effects in combined therapeutic modalities. Our study is the first to detect and evaluate the role of PROS1 in OSCC, and prompts further investigation for the involvement of PROS1 in other cancer models.

## MATERIALS AND METHODS

### Cell culture

Cells were grown in DMEM/F12 (Beit Haemek, Israel) supplemented with 10% (v/v) FBS, 100 U/ml penicillin; 0.1 mg/ml streptomycin; 2 mM L-Glutamine, at 37°C with 5% CO_2_. Cells were routinely checked for mycoplasma contamination. SCC-1 and SCC-25 cell lines originate in OSCC of the tongue. JSQ-3 originated from the nasal vestibule. Purified human PROS1 protein was purchased from Enzyme Research Laboratories (South Bend, IN). R428 was purchased from SYNkinase. SCC cell lines were transiently transfected with PROS1 siRNA (siPROS1; ON-TARGETplus, SMARTpool # L-004833; Dharmacon) or with non-targeting siRNA (siNT ON-TARGETplus, SMARTpool #D-001810; Dharmacon) using DharmaFECT transfection reagent according to the manufacturer's instructions. All pLKO.1 sh vectors were purchased from Sigma (Israel).

### Plasmids and cell transfection

Stable PROS1 knockdown (KD) was achieved in SCC1 and SCC25 cell lines using a pLKO.1 based lenriviral system (Open Biosystems). Viral vectors with different shRNA sequences targeting human *PROS1*, and empty vector (EV) or GFP sequences were used for control purposes. All pLKO.1 sh vectors were purchased from Sigma (Israel). Viral particles were assembled in 293T cells, harvested from the supernatant, and used to infect OSSC cells. Stably infected cells were selected by puromycin 0.5 μg/ml and 1 mg/ml for SCC1 and SCC25, respectively; SIGMA, Israel). At least two different sh*Pros1* constructs were used for each cell line.

### Cell viability assay

Cells were seeded overnight at a density of 2,000 cells per well in 96-well plates in DMEM containing 10% FBS and grown for the indicated time. Viable cell numbers were determined using the XTT assay kit according to the manufacturer's protocols (Beit Haemek). Each assay consisted of three replicate wells and was repeated at least three times. Data were expressed as the percentage of cells compared with control. This was calculated from the absorbance corrected for background.

### Cell proliferation assays: (BrdU, crystal violet)

Cells were seeded in 96-well plates at a density of 2-4 × 10^3^ cells/well in growth medium.

For BrdU incorporation assays, when cells reached about 40% confluence the medium was replaced with 100 microliters fresh medium containing 10 μM BrdU (Sigma) for 2 hrs, followed by one PBS wash and fixed in ice-cold methanol on ice for 30 min. Cells were washed in PBS, DNA was denatured in 2N HCl for 10 min at room temperature (RT), and neutralized with 0.1M boric acid for 10 min at RT, followed by blocking (3% FBS, 0.1% triton X-100 in PBS) for 1 hr at RT. Cells were then incubated with rat anti-BrdU (AbD serotec) at 4°C overnight (ON). The next day cells were washed 3 times in PBS and incubated with a fluorescently-tagged anti Rat antibody (Jackson Immunoresearch) for 1 hr at RT, followed by 3 PBS washes. Finally, nuclei were stained with Hoechst (0.1 μg/ml) for 5 mins, washed and mounted with Fluoromount G (Southern Biotech). Images were taken with an Olympus BX51 fluorescent microscope mounted with a DP72 camera.

For monitoring cell growth using the crystal violet assay, wells were fixed at the designated time points (24, 48, 72, 96 hr) with 100% ice-cold methanol on ice for 30 min, and stained with crystal violet solution (1% crystal violet in 2% ethanol) up to 30 min at room temperature. Cells were then washed three times with tap water and left to dry before documentation. Proliferation rates was calculated following elution of the crystal violet in 2% SDS in DDW, and the absorbance was measured at 595 nm using the iMark microplate reader (Bio-Rad). The triplicates that were fixed 24h after seeding served as a normalizing control for the number of cells seeded. All cell counts were performed in triplicates, with at least three biological repetitions.

### Cell migration assay

*In vitro* cell migration assays were performed using millicell filter chambers (8 μM pore size, Millipore). Logarithmic phase cells were resuspended in serum-free medium and 200 μl of the cell suspension (3×10^5^ cells) was added to the upper chamber. The lower chamber medium was supplemented with 21 μg/mL fibronectin (Sigma), used as a chemoattractant. Following incubation for 16 hrs at 5% CO_2_, 37°C, cells in the upper chamber were removed with a cotton swab, and migrated cells on the lower surface were stained with 2% crystal violet. Migration assays were performed in triplicates or sextuplicates. The membranes were allowed to dry, documented using an Olympus DP72 camera mounted on an Olympus inverted microscope (CKX41). Migration rate was then calculated following elution of the crystal violet as described above.

### Quantitative real-time PCR (qPCR)

Cells were harvested, washed once with PBS and RNA was isolated with TRIZOL (Sigma). cDNA was synthesized (qScript cDNA synthesis kit, Quanta Biosciences). Real-Time reactions were performed in triplicate using KAPA SYBR FAST qPCR Kit (2X) (KAPA-Biosystems) following manufacture's instructions on a CFX96 Real Time PCR Cycler (Bio-Rad). The reactions were normalized to β*-actin* or *Gapdh*, using the ΔΔ threshold cycle (Ct) method. Specificity of the primers was checked by dissociation curves. Primer sequences are provided in Supplementary Table 1.

### Western blot

Cells were harvested, washed once with PBS, and whole cell lysates were prepared in lysis buffer (50 mM HEPES pH 7.5, 150 mM NaCl, 10 mM EDTA, 10% glycerol, 1% Triton X-100, 1 mM Na_3_VO_4_, 1 mM NaF). Phosphatase and protease Inhibitor cocktails (Sigma) were added freshly. Protein was determined using the BCA method (Thermo Fisher) following manufacturer's instructions. Equal protein amounts were separated by SDS-PAGE on 8% or 10% Tris-glycine, then transferred to PVDF membranes (Millipore), blocked in 5% skim milk (BD Difco) for 1 hour at RT, and reacted with primary antibody (as listed in Supplementary Table 1) overnight at 4°C. Next day the membrane was washed 3 times in 1X TBST for 10 min at RT. Incubation with secondary HRP-conjugated antibody in blocking buffer for 1 hour at RT was followed by 4 washes in TBST and blots were reacted with ECL substrate (Western blot detection kit, Advansta). Images were captured using a ChemiDocTM MP Image System (Bio-Rad), and band quantification was done using the ChemiDoc software.

### Anchorage-independent growth

Colony formation in soft agar was assayed in triplicate by plating 30,000 of the indicated cells in a layer of 0.35% (w/v) agar in assay medium, on top of a 0.7% (w/v) agar layer. Plates were incubated at 37 °C and 5% (v/v) CO2 for 14 d, and the medium was replaced every 4 d. Colonies were stained using 0.005% (w/v) Crystal Violet solution, and an image of the whole well was acquired using an Olympus SZ10 stereoscope mounted with a DP72 camera. Colonies were counted using ImageJ software (http://imagej.nih.gov/ij/), and the mean ± SEM were calculated average number of colonies per well was calculated.

### Wound-healing assay

Indicated cells were seeded in a 6 or 96 well plate until formation of a confluent monolayer, and a “wound” was created by scratching the monolayer with a p10 sterile pipette tip. Cells were washed with PBS and growth medium was replaced with fresh growth medium. The wound was photographed (× 40 magnification) after matching a reference point in a phase-contrast microscope and wound area was measured using the T-scratch software. Alternatively, 4×10^4^ cells were plated in 96 wells and allowed to adhere overnight. The 96 well plate was inserted into Incucyte Zoom system (Essen Biosciences), which performed automated scratches. The migration was monitored by phase imaging with an acquisition every 2 hrs, for 24 hrs. The Incucyte Zoom analysis software automatically calculated the cell coverage area. All wound-healing experiments were performed in between 3-6 replicates per experiment.

### Xenograft tumor formation in mice

Pools of stably transformed cells expressing the control (EV) or sh*Pros1* constructs (2 × 10^6^ cells per site in 100 μl of serum-free media using a 26-gauge needle) were injected into the flank of NOD/SCID mice. Tumor growth was monitored by caliper measurement. Veterinary care was provided to all animals by the Hebrew University animal care facility staff in accordance with AAALAC standard procedures and as approved by the Hebrew University Ethics committee.

### Cytokeratin, Ki-67 and TUNEL staining on paraffin sections and cultured cells

Paraffin-embedded sections (5 microns thick) of the indicated tumors were processed for immunohistochemistry according to standard protocols. Briefly, sections were deparaffinized in xylenes followed by rehydration in ethanol series, and equilibrated in PBS. Antigen retrieval was performed in 10 mM Na-citrate buffer (pH 6); 0.05% tween-20 in a pressure cooker for 20 mins, washed in PBS several times, blocked and incubated with anti-Ki-67 overnight at 4°C. The sections were washed the next day, incubated with a fluorescently-conjugated antibody at room temperature for 2 hrs, stained for Hoechst and mounted with Fluoromount (Southern Biotech). Images were taken using a C2 Nikon confocal microscope. For the TUNEL assay, slides were deparaffinized and processed with the *In Situ* Cell Death Detection kit – TMR red (Roche Diagnostics, GmBH) according to manufacture's instructions.

For cytokeratin immunohistochemistry on cultured cells: all solutions were prepared in PBS with Ca2^+^ and Mg2^+^. Cells were washed and fixed in 4% paraformaldehyde for 10 mins at RT, washed and permeabilized with 0.5% NP-40 in PBS for 10 mins at R.T., followed by another PBS wash. Cells were then blocked in 20% FCS in PBS for 15 mins at R.T in a humid chamber. Primary antibody was added at the indicated dilution (see Supplementary Table 2) for 1 hr at R. T, washed and incubated with secondary antibody 30 min at R.T. Cells were then Hoechst-stained, mounted and observed. Images were taken using a C2 Nikon confocal microscope.

### Drug treatment

5000 cells/well were seeded in triplicates or sextuplicates on 96 well plates in full growth medium. Cells were allowed to adhere for 24 hours, when a triplicate for each cell line was fixed and served for cell number normalization. For all other triplicates, the medium was replaced with fresh growth medium containing increasing concentrations of R428. Cells were further incubated for another 48 hours in the presence of R428, fixed and quantified using the crystal violet method. Cell number is presented as fold (in %) over the number of cells seeded.

### Immunohistochemistry and histopathological analysis

Primary tumors were harvested and immersion-fixed in 4% Paraformaldehyde, processed, embedded in paraffin and sectioned. For PROS1 immunostaining, Heat induced antigen retrieval was performed in Citrate buffer (0.1 M Na-Citrate (pH=6.0); 0.05% Tween-20 in PBS), the sections blocked (5% NGS; 0.05% Triton X-100 in PBS) for 1 hr at room temperature, and incubated overnight at 4°C with anti -human PROS1 antibody (DAKO). The sections were thoroughly washed in PBS and incubated with secondary antibody. For HRP-conjugated secondary antibody, the colorimetric reaction was developed using DAB substrate, followed by Hematoxylin counter staining. For Fluorescently-conjugated secondary antibodies, slides were mounted with Fluoromount G (Southern Biotech). Cytokeratin staining was performed by the Hadassah Pathology department with an automated Benchmark XT IHC system. Anti-cytokeratin 5/6 (Invitrogen Cat # 18-0267; 1:100) IHC was performed using the standard CC1 antigen retrieval protocol (64 mins) followed by anti CK5/6 iantibody incubation (40 min at 37°C), and ultraview universal DAB. For CK8/18 (Dako; Cat # M3652; 1:50) and anti CK19 (Dako M0888; 1:75), tissue was pre-treated with Protease I (8 min) followed by antibody incubation at 37°C and DAB (Ventana, Tuscon, AZ). Anti CK8/18 was incubated for 40 min and processed with ultraview DAB; anti CK19 was incubated for 32 min and processed with i-view DAB. Images were taken using Olympus BX51 microscope mounted with an Olympus DP72 camera.

For pathological evaluation, tumors sections were stained with Haematoxylin and Eosin, and scoring was performed using criteria-based routine pathological methods based on a modification of the conventional grading system [24] by an oral pathologist who was blinded to the experimental design. Briefly, the histologic malignancy grading included cytoplasmic differentiation (keratinization, pearl structures), nuclear polymorphism and differentiation (mitoses) and severity of invasiveness. Vascular/Lymphatic invasion and lymphocytic infiltration were occasionally observed but were not graded in this study.

### Statistics

Data represent mean ± SEM of experiments. At least three independent experiments were performed per assay. Significance was determined using a Student's t test,

The results of quantitative studies are reported as mean ± SEM. The SEM was calculated on the basis of the number of independent experiments. Differences were analyzed by the Student t test considering P<0.001 as extremely significant (***), P<0.01 as highly significant (**) and P<0.05 as statistically significant (*). P < 0.05 were regarded as significant.

## SUPPLEMENTARY FIGURES AND TABLES


