# Supplementary material for: Protein S drives oral squamous cell carcinoma tumorigenicity through regulation of AXL
Source: Oncotarget. 2017 Jan 19;8(8):13986–4002. doi: 10.18632/oncotarget.14753 (PMC5355156; doi:10.18632/oncotarget.14753)
Supplement: Supplementary file 1 [file oncotarget-08-13986-s001.pdf]

Protein S drives oral squamous cell carcinoma tumorigenicity through regulation of AXL

SUPPLEMENTARY FIGURES AND TABLES

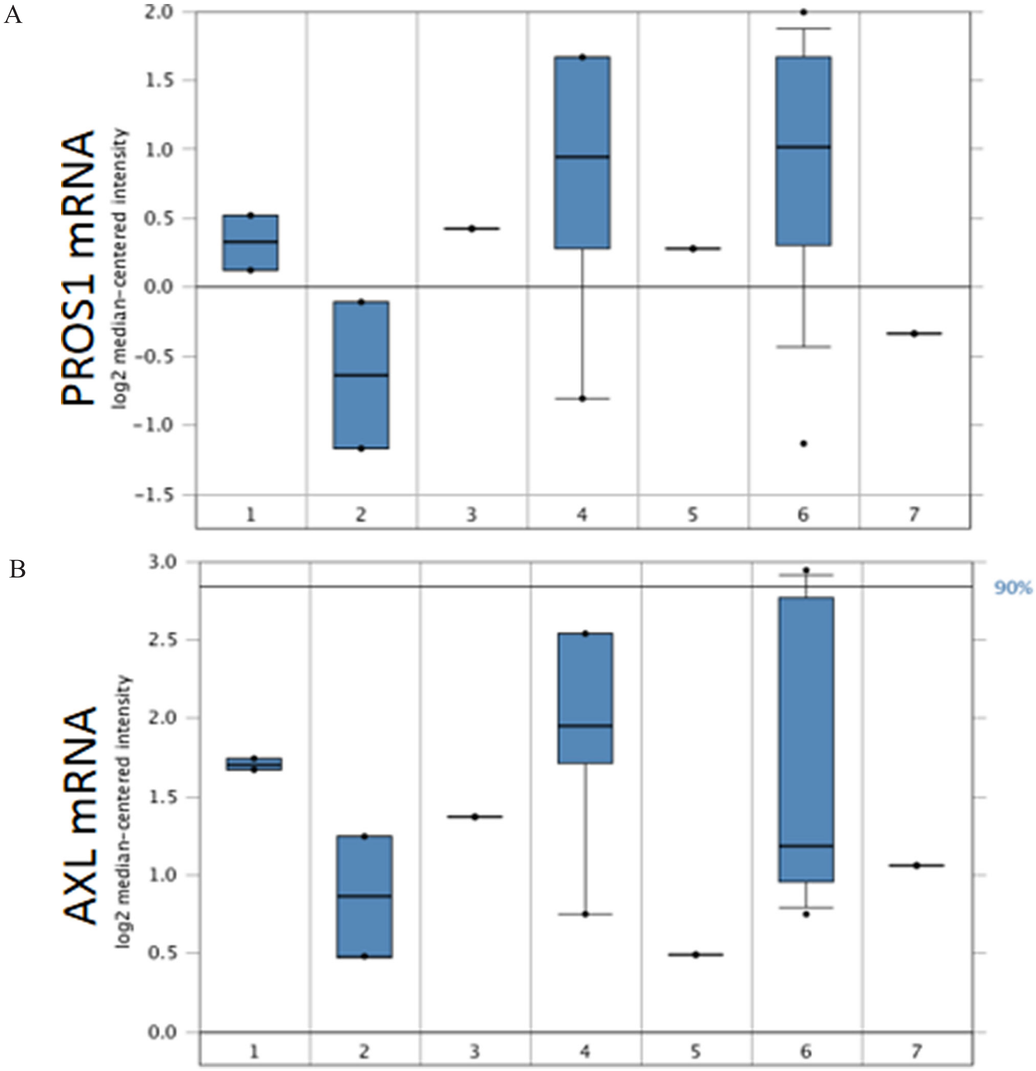

Supplementary Figure 1: mRNA expression of PROS1 A. and AXL B. in oral cancers. mRNA expression levels of PROS1in Oral Squamous Cell Carcinomas derived from different sites in the oral cavity (27 samples). Analyses was done using the Oncomine database (www.oncomine.org). Data are derived from O'Donnell Oral Database [22].

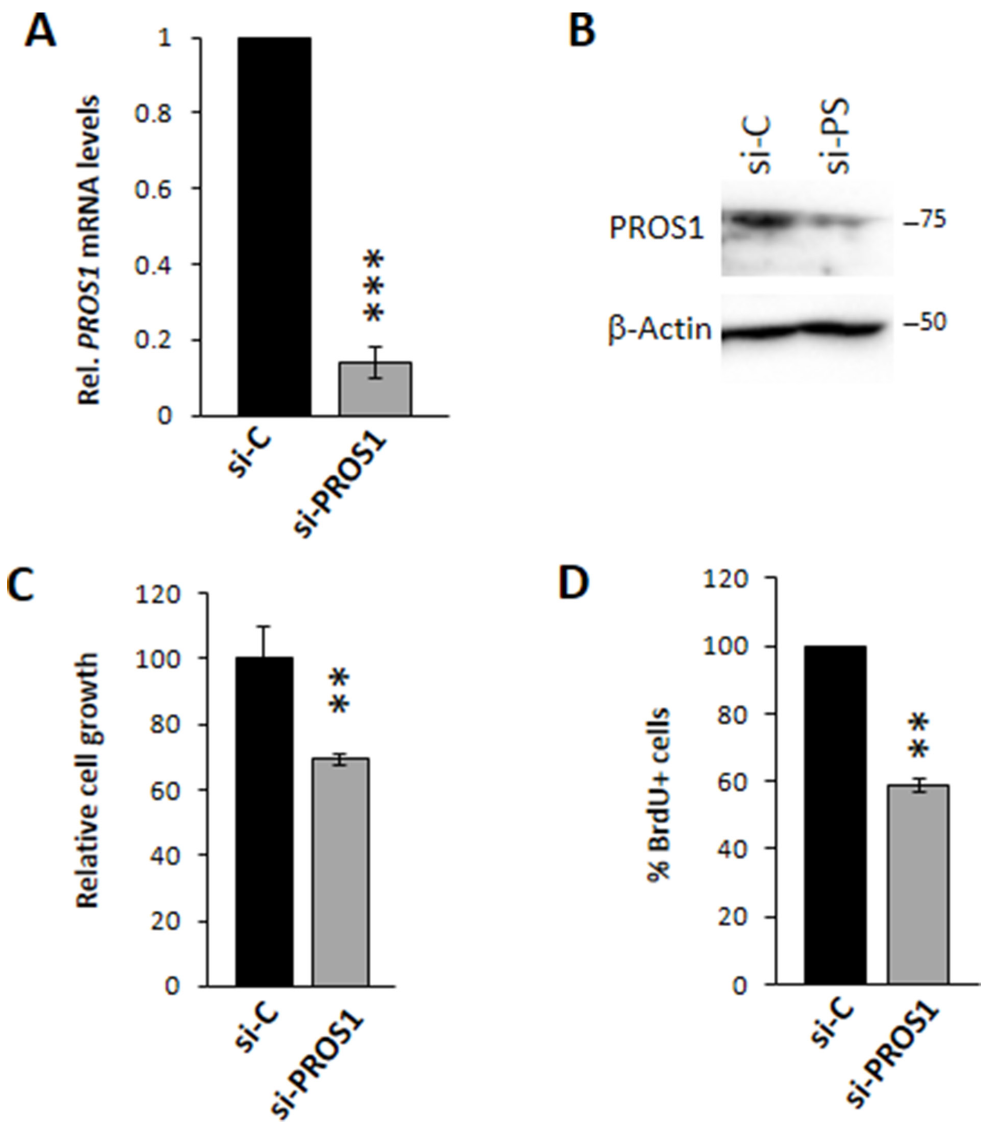

Supplementary Figure 2: si-mediated inhibition of *PROS1* results in decreased cell proliferation. **A.** Effective knockdown of *PROS1* in SCC-1 cells following si-mediated RNA inhibition as detected by RT-qPCR 48 hrs following si-*PROS1* treatment. Results are representative of three independent experiments. ***$P<0.0001$. **B.** Inhibition of PROS1 protein levels following si-*PROS1* treatment, as detected by western blot. Representative data of one of three independent experiments is shown. Actin serves as a loading control. **C.** Inhibition of cell proliferation following si-*PROS1*. Proliferation was measured by crystal violet absorbance 24 hours after treatment, and is presented as relative growth compared to EV-treated cells. Cell counts were normalized to the number of cells seeded. Results are representative of three independent experiments, with each condition performed in triplicate. **$P=0.007$. **D.** Inhibition of BrdU incorporation following si-*PROS1* treatment. Results are presented as % of BrdU+ cells, following anti-BrdU immunocytochemistry. Results are representative of three independent experiments, with each condition performed in triplicate. **$P=0.01$.

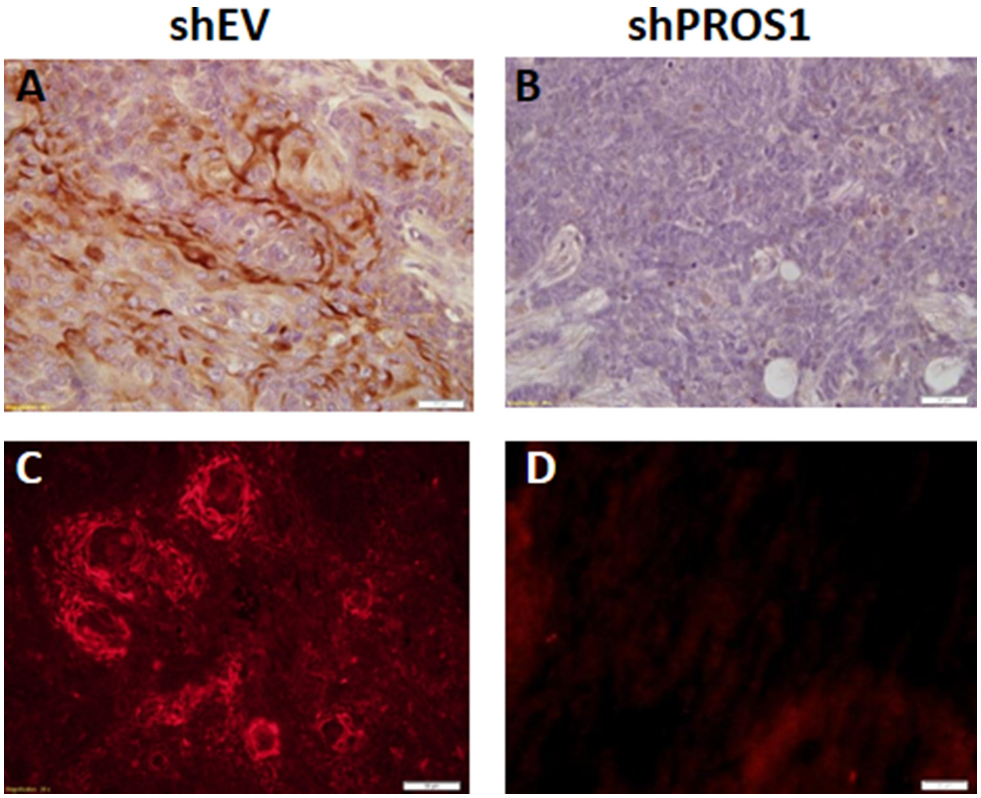

Supplementary Figure 3: PROS1 immunoreactivity in xenograft tumors. A-D. PROS1 immunoreactivity is detected in control-treated (sh-EV, A, C) tumors, but not in sh-PROS1-treated tumors (B, D). Tumors were sectioned and stained for PROS1 (brown in A, red in C) following antigen retrieval, and counterstained with hematoxylin. Strong PROS1 protein signal is detected in cells of the basal layer of tumor nests, surrounding OSCC typical keratin pearls. PROS1 immunoreactivity is shown following colorimetric (A, B) and fluorescent detection, emphasizing PROS1 expression in the outer basal cells of the tumor nests. Bar: A, B = 20 µM; C, D = 50 µM.

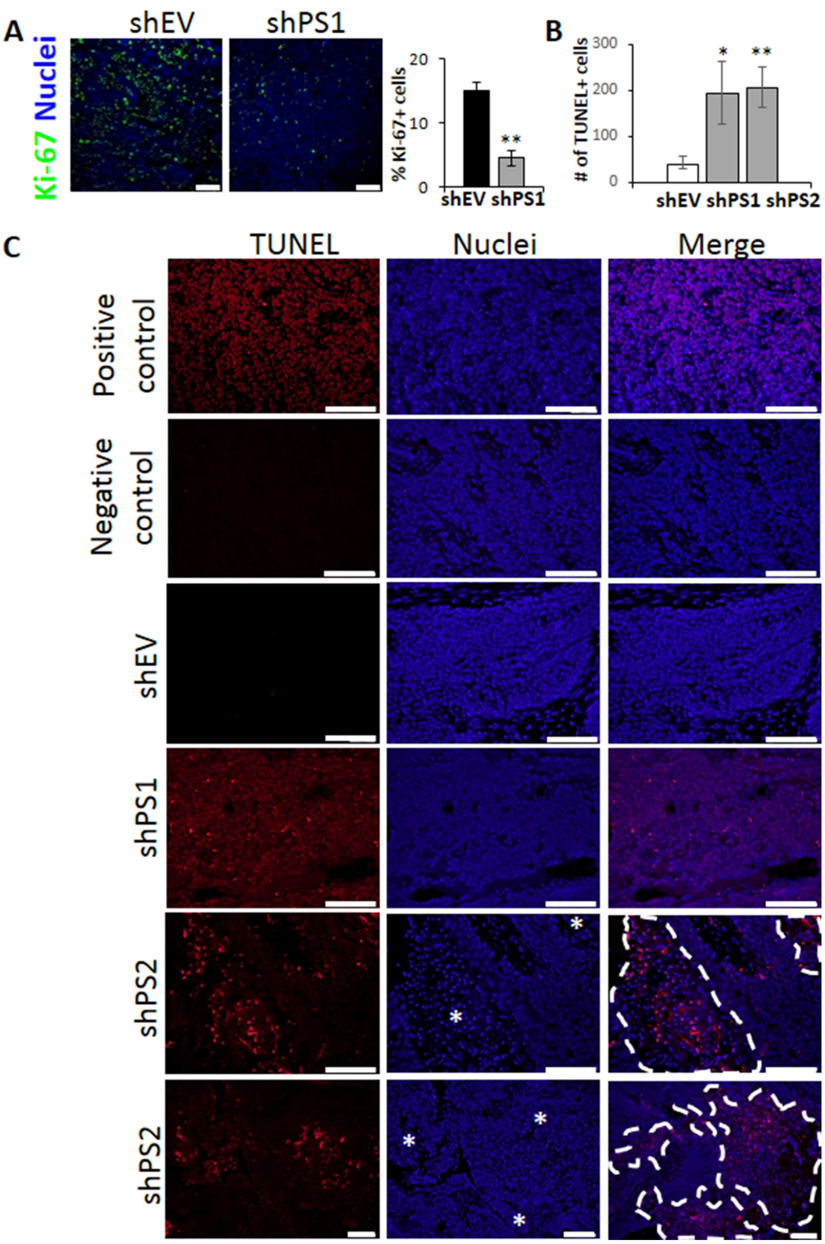

Supplementary Figure 4: Decreased proliferation with enhanced apoptosis in PROS1-KD tumors. A. Inhibition of PROS1 alters the numbers of Ki-67+ cycling cells. Representative images of anti-Ki-67 immunoreactivity (green) in xenograft tumors from SCC-1-shEV (control, left) and PROS1-kd (SCC1-shPS1, right) xenografts. Nuclei are stained with Hoechst (blue). Bars represent the mean values ± SEM from a minimum of three different tumors per group. At least three sections from different locations were analyzed per tumor, with a minimum of five different fields scored per section. The percentage of the stained area, normalized to nuclei staining, was calculated using FIJI software. **P=0.0042. Bars = 100 µM. **B.** PROS1 knockdown induces apoptosis in tumors. Quantification of TUNEL-positive cells in shEV, shPS1 and shPS2 xenografts. Bars represent the mean values ± SEM from four different tumors per group. Quantification was performed using FIJI software. Five different fields were scored per section; three sections from different locations were analyzed per tumor. *P=0.024; **P=0.0015. **C.** Representative images of the TUNEL reaction performed on xenograft sections. Images of TUNEL reactivity (red, left panels), Hoechst-stained nuclei (Blue, middle panels) and merged images (right panels) are shown. Positive and negative controls are shown at the top rows, respectively. A lower power magnification is presented at the bottom row to demonstrate the proximity of numerous TUNEL-positive apoptotic foci, delineated by the dashed line and marked by asterisks. Areas dominated by pyknotic nuclei undergoing nuclear condensation are visualized by Hoechst staining, and are marked with an asterisk. These pyknotic centers are also rich in TUNEL-positive cells (red). Bars = 100 µM.

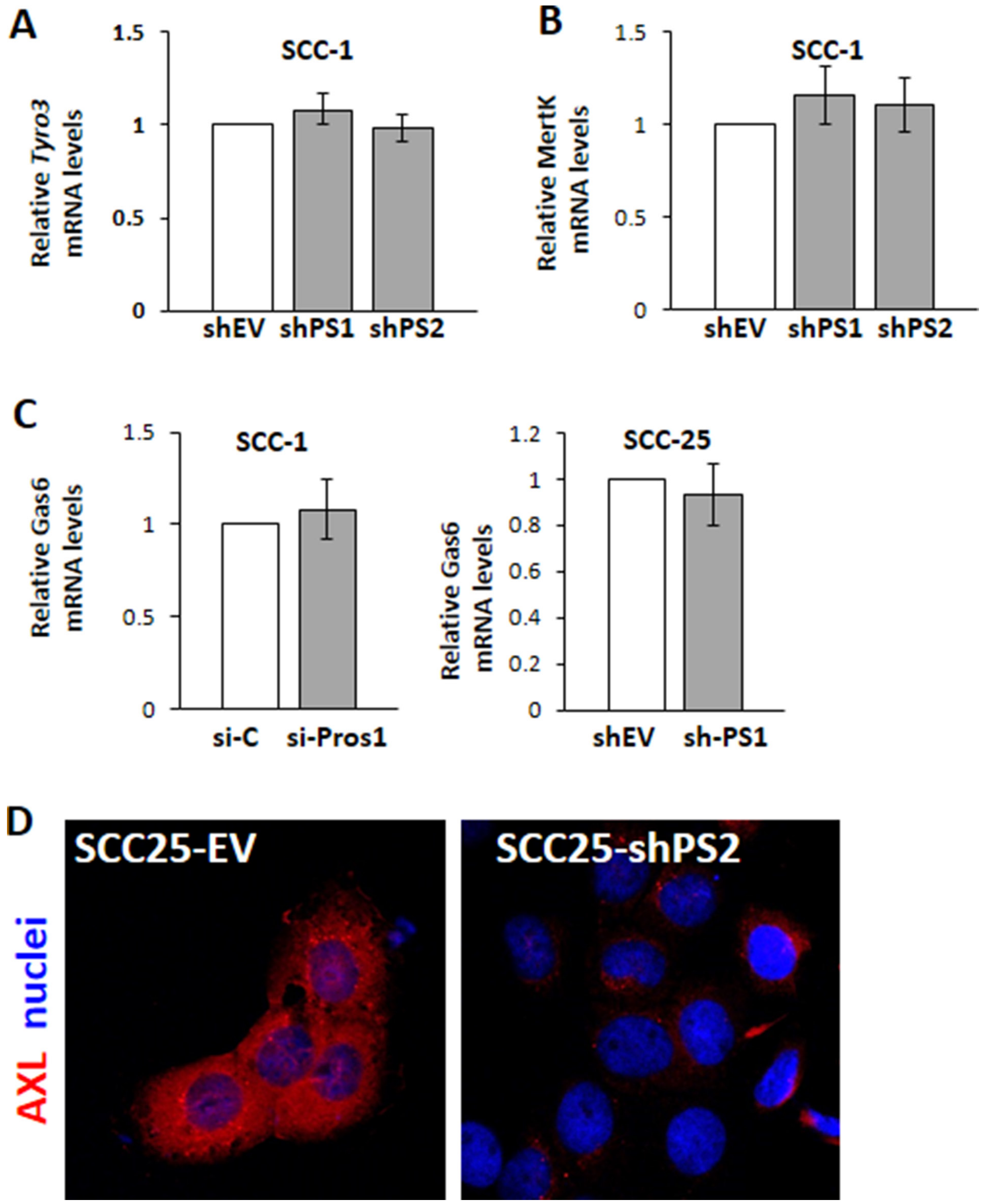

Supplementary Figure 5: Tyro3, Mertk and Gas6 levels are not affected by PROS1 knockdown. A-C. Relative mRNA levels of *TYRO3* (A), *MERTK* (B) and *GAS6* (C) were unaffected by PROS1 kd either by lentiviral mediated knockdown (shPS1 and shPS2) or by transient RNAi. Mean relative expression ± SEM from three independent experiments are shown. **D.** Additional validation for AXL protein being downregulated by PROS1-knockdown. Immunocytochemistry for AXL (red) is shown, indicating decreased AXL density on the cell surface. Nuclei are stained with Hoechst (blue).

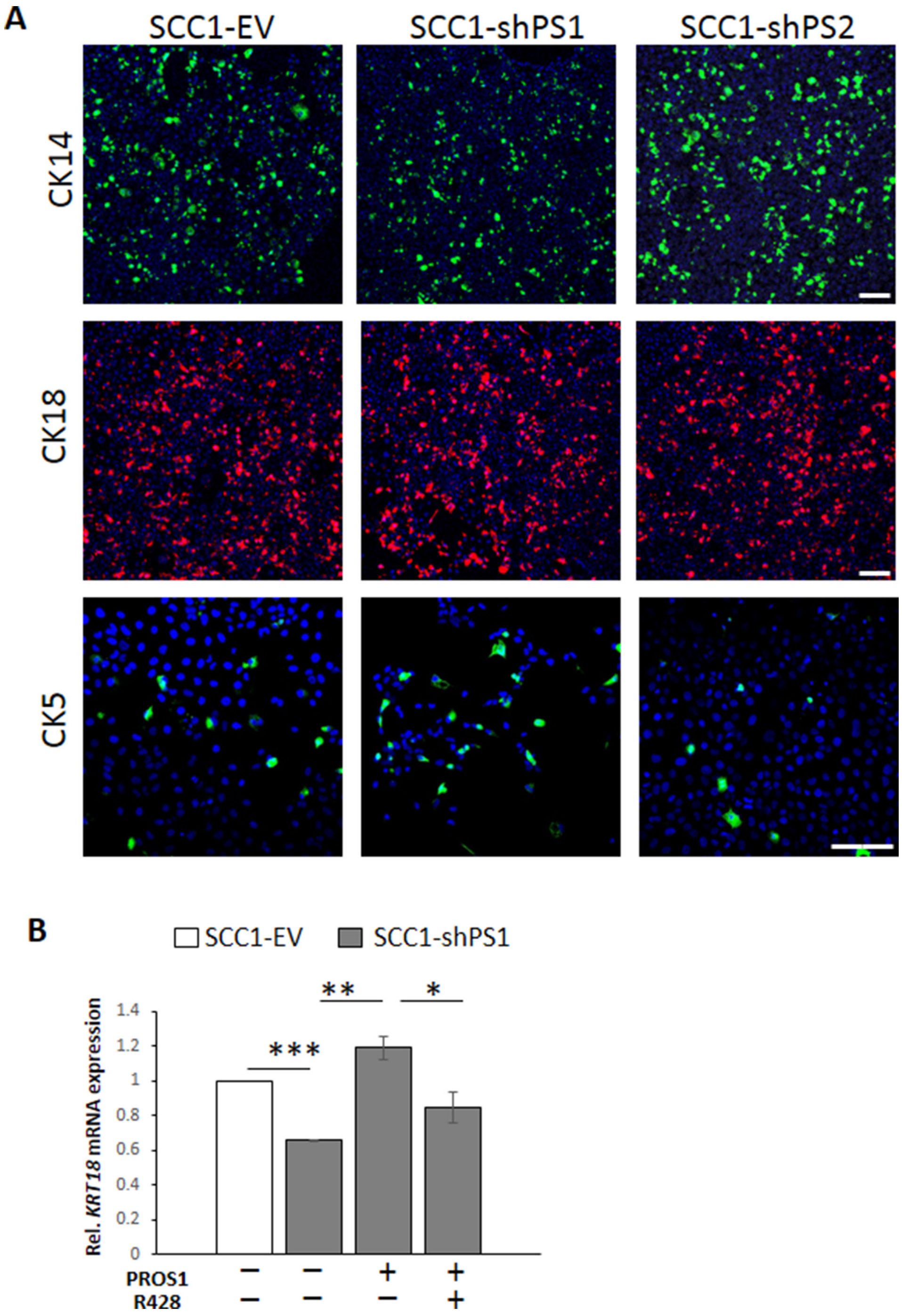

Supplementary Figure 6: Cytokeratin mRNA and protein are differentially regulated in culture. Cytokeratin protein expression is affected by cytoarchitecture. **A.** Cultured SCC1 control and knockdown lines were assessed for cytokeratin expression by immunocytochemistry. Representative imunofluorescent images of CK14 (top), CK18 (center) and CK5 (bottom) in SCC1-EV (left), shPS1 (middle) and shPS2 (right) are shown. Bar = 100 µM. **B.** mRNA levels of KRT18 are responsive to PROS1. Quantification by RT-qPCR of KRT18 expression levels in SCC1 control and shPS1 lines treated or not with PROS1 (28 nmol/L) or PROS1 and R428 (50 nmol/L). Relative expression was normalized to *GAPDH*. Representative data of one of three independent experiments is shown. ***P<0.0001, **P<0.01; *P<0.05.

Supplementary Table 1: Primer sequences table

Gene	Forward primer	Reverse primer
hPROS1	ACT TGC CGT CTT GGA CAA AGC A	AGG CAT TCA CTG GTG TGG CAC T
hAXL	GTT TGG AGC TGT GAT GGA AGG C	CGC TTC ACT CAG GAA ATC CTC C
hTYRO3	GCA AGC CTT TGA CAG TGT CAT GG	GTT CAT CGC TGA TGC CCA AGC T
hMERtk	CAG GAA GAT GGG ACC TCT CTG A	GGC TGA AGT CTT TCA TGC ACG C
hβ-actin	TCC TTC CTG GGC ATG GAG T	AGC ACT GTG TTG GCG TAC AG
hGAPDH	TCC ACT GGC GTC TTC ACC	GGC AGA GAT GAT GAC CCT TTT
KRT18	ATC TTG GTG ATG CCT TGG AC	CCT GCT TCT GCT GGC TTA AT
hFAT1-set 1	GAG TGG TTT CGT CCA AG	TGG AGT CTG GTG GTT GAT GA
hFAT1-set 2	TAG ATG GCA ACC AAG GAA GC	CGT GAG CGT GTA ACC TGA AA
hFAT1-set 3	TTT TAA CCC CGA GTC TGT GC	GCT GTC AGA AGG GGA GTT TG

Supplementary Table 2: Antibodies used in this study

Primary antibody	Antibody dilution	Manufacturer	Cat. number
Rat anti BrdU	1:100	AbD serotec	OBT0030
Rb anti hPROS1	1:1000	DAKO	A0384
Gt anti AXL	1:1000	Santa Cruz	SC-1096
Gt anti Actin	1:1000	Santa Cruz	SC-1616
Rb anti AXL (C89E7)	1:1000	Cell Signaling	8661
Anti-cytokeratin 5/6	1:100	Invitrogen	18-0267
Anti-cytokeratin 5	1:150	Abcam	52635
Anti-cytokeratin 8/18	1:50	DAKO	M3652
Anti-cytokeratin 19	1:75	DAKO	M0888
Anti-cytokeratin 18	1:50	Thermo Scientific	Ms-142-P
Anti-cytokeratin 14	1:50	Thermo Scientific	Rb-9020-P
Anti Ki-67	1:100	Abcam	AB16667
